# The prognostic value and therapeutic targeting of myeloid-derived suppressor cells in hematological cancers

**DOI:** 10.3389/fimmu.2022.1016059

**Published:** 2022-10-11

**Authors:** Rong Fan, Nathan De Beule, Anke Maes, Elke De Bruyne, Eline Menu, Karin Vanderkerken, Ken Maes, Karine Breckpot, Kim De Veirman

**Affiliations:** ^1^ Department of Hematology and Immunology-Myeloma Center Brussels, Vrije Universiteit Brussel, Brussels, Belgium; ^2^ Department of Clinical Hematology, Universitair Ziekenhuis Brussel, Brussels, Belgium; ^3^ Center for Medical Genetics, Vrije Universiteit Brussel, Universitair Ziekenhuis Brussel, Brussels, Belgium; ^4^ Laboratory for Molecular and Cellular Therapy, Department of Biomedical Sciences Vrije Universiteit Brussel, Brussels, Belgium

**Keywords:** hematological cancers, myeloid-derived suppressor cells, immunotherapies, multiple myeloma, leukemia, lymphoma

## Abstract

The success of immunotherapeutic approaches in hematological cancers is partially hampered by the presence of an immunosuppressive microenvironment. Myeloid-derived suppressor cells (MDSC) are key components of this suppressive environment and are frequently associated with tumor cell survival and drug resistance. Based on their morphology and phenotype, MDSC are commonly subdivided into polymorphonuclear MDSC (PMN-MDSC or G-MDSC) and monocytic MDSC (M-MDSC), both characterized by their immunosuppressive function. The phenotype, function and prognostic value of MDSC in hematological cancers has been intensively studied; however, the therapeutic targeting of this cell population remains challenging and needs further investigation. In this review, we will summarize the prognostic value of MDSC and the different attempts to target MDSC (or subtypes of MDSC) in hematological cancers. We will discuss the benefits, challenges and opportunities of using MDSC-targeting approaches, aiming to enhance anti-tumor immune responses of currently used cellular and non-cellular immunotherapies.

## 1 Introduction

The tumor microenvironment is a complex and dynamic network of distinct cell types (1–3). The composition of the environment is variable between different tumor types; however, it typically includes stromal cells, blood vessels, immune cells and extracellular matrix (4). Myeloid-derived suppressor cells (MDSC), tumor associated macrophages (TAM) and regulatory T-cell (Treg) are major components of the microenvironment and are critical drivers of immunosuppression, creating a tumor-promoting and drug resistant niche (5, 6).

MDSC are a heterogeneous population of immature myeloid cells and are generated in the bone marrow (BM) by myelopoiesis (7). Under healthy conditions, the precursor cells can terminally differentiate into mature dendritic cells, granulocytes or macrophages. However, in pathological circumstances including cancer, the differentiation of precursor cells is partially blocked, leading to an accumulation of an immature myeloid cell population, defined as MDSC (8, 9). MDSC are known to accumulate during cancer progression and promote tumor immune escape through multiple mechanisms including (i) the expression of enzymes [e.g., arginase (Arg), nitric oxide synthase (NOS), indoleamine 2,3-dioxygenase (IDO)], (ii) the release of reactive oxygen species (ROS), (iii) sequestering of cystine (↓ extracellular pool of cysteine), (iv) the interaction and stimulation of other immunosuppressive cell types (e.g., Treg) and (v) the secretion of immunosuppressive cytokines (e.g., IL-6, IL-10, TGF-β) (10–13).

In hematological malignancies, the presence and accumulation of MDSC is often correlated with a poor prognosis, however the optimal strategy to specifically eliminate MDSC or alter their suppressive function remains challenging (14). Immunotherapy emerged as one of the most promising treatment options for almost all types of hematological cancers and is primarily focused on the modulation/stimulation of T-cell using monoclonal antibodies, bispecific T-cell engagers, cell therapies, vaccines and immune checkpoint inhibitors (e.g., PD-1-, LAG-3-, CTLA-4-blocking antibodies) (15). In this regard, therapeutic strategies to tackle immunosuppressive cell types (including MDSC, TAM and Tregs) became an interesting option to increase anti-tumor immune responses and overcome the occurrence of drug resistance to currently used or investigated cancer immunotherapies.

## 2 MDSC phenotype and prognostic value in hematological cancers

MDSC are commonly subdivided into two groups: monocytic MDSC (M-MDSC) and granulocytic (or polymorphonuclear) MDSC (G-MDSC). The phenotype and morphology of M-MDSC is very similar to monocytes, G-MDSC and neutrophils also share common characteristics (e.g., arginase-mediated arginine depletion) (16–18). Despite the morphological and phenotypical similarities, functional differences between steady-state neutrophils and G-MDSC are described including a higher activity of arginase, myeloperoxidase (MPO), and ROS; reduced expression of CD16 and CD62L; and less granules in G-MDSC compared to neutrophils (17, 19). In recent years, it became clear that M-MDSC and G-MDSC also utilize distinct mechanisms to suppress the immune system. M-MDSC hamper T-cell responses in a STAT1/3- and iNOS-dependent manner, which is associated with increased NO and immunosuppressive cytokine production (IL-10, TGF-β). The effect of G-MDSC, on the other hand, is attributed to an antigen-specific induction of T-cell tolerance by STAT3 activity and increased expression of Arg-1, ROS, peroxynitrite and prostaglandin E_2_ (8).

In humans, the distinction between MDSC and monocytes/neutrophils can be made based on density gradient and phenotypic markers (e.g., expression HLA-DR), however the distinction between these subtypes in mice is much more challenging and therefore the nature and uniqueness of the MDSC populations continues to be a matter of debate. In murine models, MDSC are phenotypically defined as CD11b^+^GR1^+^ and further subdivided into CD11b^+^Ly6G^−^Ly6C^low^ for M-MDSC and CD11b^+^Ly6G^+^Ly6C^low^ for G-MDSC. In humans, both subtypes are distinguished based on the following phenotypic markers: CD11b^+^CD14^+^CD15^−^CD33^+^HLA-DR^−/low^ for M-MDSC and CD11b^+^CD14^−^CD15^+^CD33^+^(CD66b^+^) for G-MDSC (17, 18). More recently, in humans, a third “early-stage” MDSC subset (eMDSC) has been identified, characterized as Lin^−^ (CD3/14/15/19/56) HLA-DR^−^CD33^+^. This subset comprises immature progenitor and precursor cells with colony-forming activity, however its exact function and contribution to immune suppression remains unclear (20). Various reviews described the presence and immunosuppressive function of MDSC in hematological malignancies, however below we aimed to provide a brief and structured overview about the main findings on MDSC subsets and their prognostic value in different hematological cancers as this is particularly important in the context of therapeutic strategies (**Table 1**) (14, 20, 47–50).

**Table 1:** Summary of MDSC representative phenotype and their prognostic role in different hematological cancers.

Diseases		Source	MDSC subgroups/phenotype definition	Clinical finding	Ref
Leukemia	AML	PB BM	M-MDSC: CD11b^+^HLA-DR^-^CD14^-/+^CD33^+^CD15^-^ G-MDSC: CD11b^+^HLA-DR^-^CD14^-^CD33^+^CD15^+^	Higher MDSC level in PB and BM of AML patients VS. HD.	(21)
		PB	M-MDSC: CD14^+^HLA-DR^low/-^	Higher circulating M-MDSC frequencies in CD14^+^ monocytes and PBMC VS. HD (p < 0.01).	(22)
		PB	eMDSC: Lin^-^(CD3/14/15/19/56)HLA-DR^-^CD33^+^	Unknown	(23)
		BM	MDSC: CD33^+^CD11b^+^HLA-DR^low/neg^	Significantly increased MDSC in BM (p < 0.01).	(24)
	CML	PB	M-MDSC: CD14^+^HLA-DR^-^ G-MDSC: CD11b^+^CD33^+^CD14^-^HLA-DR^-^	MDSC levels were increased at diagnosis and returned to normal levels after therapy (p < 0.001, p < 0.0001).	(25)
		PB	MDSC: CD11b^+^ CD14^-^CD33^+^	PB MDSC levels were increased in samples from Sokal high-risk patients (p < 0.05).	(26)
	B-ALL	PB BM	M-MDSC: CD45^+^CD19^-^HLA- DR^-^CD11b^+^CD33^+^CD14^+^ G-MDSC: CD45^+^CD19^-^HLA- DR^-^CD11b^+^CD33^+^CD15^+^	G-MDSC were significantly elevated in PB and BM vs. age-matched HD (p < 0.05, p < 0.01). G-MDSC levels correlated positively with clinical therapeutic responses and B-ALL disease prognostic markers.	(27)
		PB	MDSC: LinHLA-DR^-^CD33^+^CD11b^+^	MDSC levels significantly increased in early diagnosed B-ALL patients VS. HD.	(28)
	CLL	PB	M-MDSC: CD14^+^HLA-DR^low/-^	The M-MDSC were upregulated in patients (p < 0.0001) and were correlated with CLL tumor progression, poor prognosis, and correlated with the presence of CD4^+^ T and CD5^+^CD19^+^ cells.	(29)
		PB	M-MDSC: CD14^+^CD11b^+^CD15^-^HLA-DR^low/-^	M-MDSC were increased in PB of CLL Patients and correlated with The Rai Stage (p < 0.001), and a close association with unfavorable prognostic markers.	(30)
		PB	M-MDSC: CD14^+^CD11b^+^CD15^-^HLA-DR^low/-^	Higher median percentage of M-MDSC with IL-10 or TGF-1 expression in CLL patients than in HD (p < 0.001, p < 0.0001).	(31)
		PB	M-MDSC: HLA-DR CD11b^+^CD33^+^CD14^+^ G-MDSC: HLA-DR^low^CD11b^+^CD33^+^CD15^+^	Higher numbers of G-MDSC in patients correlated with different Th- subsets, and were more strongly associated with a poor clinical course than M-MDSC.	(32)
Lymphoma	DLBCL	PB	M-MDSC: CD14^+^HLA-DR^-^ G-MDSC: Lin^-^CD123^-^HLA-DR^-^CD33^+^CD11b^+^	Increased M-MDSC and G-MDSC populations in whole blood VS. HD (p = 0.001, p = 0.01). M-MDSC were correlated with the IPI and EFS (p = 0.034, hazard ratio = 0.19).	(33)
		PB	M-MDSC: CD14^+^HLA-DR^low/-^	Increased frequency of M-MDSC was found in ND vs. HD (p < 0.01) and associated with tumor progression in patients. (ND vs. Rel VS. Rem, p < 0.05, p < 0.01).	(34)
	HL	PB	MDSC: CD11b^+^CD33^+^CD14^-^CD34^+^HLA-DR^-^ M-MDSC: CD14^+^HLA-DR^low/-^ G-MDSC: CD11b^+^CD33^+^CD14^-^HLA-DR^-^Lin^-^	All MDSC subsets (immature MDSC, G-MDSC, M-MDSC) were higher in patients VS. HD (p = 0.03, p = 0.02, p 0.04), and higher MDSC percentages were present in non-responders. CD34^+^ immature MDSC were predictive for a short PFS in HL patients (p = 0.03).	(35)
	B-NHL	BM	M-MDSC: CD14^+^CD33^+^HLA-DR^-^ G-MDSC: CD10^-^HLA-DR^low/-^	Differences in M-MDSC (ND, Rem and Rel of B-NHL patients vs. HD, p < 0.0001, P < 0.001, p < 0.001). G-MDSC% was increased in PB (ND and Rem and Rel of B-NHL patients vs. HD, p <0.0001, p < 0.0001, p < 0.0001).	(36)
Multiple Myeloma		PB	M-MDSC: CD14^+^HLA-DR^low/-^	Increased level of MDSC in patients with MM at diagnosis VS. HD (p < 0.05).	(37)
		PB BM	M-MDSC: CD14^+^HLA-DR^low/-^	M-MDSC of ND MM patients were increased in PB and BM vs. HD (p < 0.01), and were associated with MM progression and response to therapy (ND and Rem and Rel of MM patients VS. HD, p < 0.01).	(38)
		PB BM	M-MDSC: CD11b^+^CD14^+^HLA-DR^low/-^ G-MDSC: CD11b^+^CD33^+^HLA-DR^low/-^CD14^-^ CD15^+^	PB M-MDSC show correlation with serum creatinine, lactate dehydrogenase, and β-microglobulin and inverse correlation with hemoglobin level PB M-MDSC of patients with progressive disease showed higher levels than those of patients at diagnosis and in complete response (p = 0.003 and 0.026, respectively). BM M-MDSC levels were higher in patients with progressive disease than those in patients at diagnosis (p = 0.007). PB M-MDSC > 0.3%) at diagnosis had an independent adverse prognostic impact on OS.	(39)
		PB	M-MDSC: CD14^+^HLA-DR^low/-^ eMDSC: CD11b^+^Lin^-^(CD3/14/15/19/56)HLA- DR^-^CD33^+^	In the pre-ASCT analyses, lower M-MDSC (median) were associated with a longer time to progression (TTP) (p < 0.001). Pre-ASCT M-MDSC more strongly inhibited the *in vitro* cytotoxic effect of mephalan compared with pre-ASCT eMDSC (p < 0.01).	(40)
		PB	M-MDSC: G-MDSC: CD10^-^HLA-DR^low/-^	Higher G-MDSC in PB of ND and Rel VS. HD (p = 0.03, p < 0.001).	(41)
		PB BM	M-MDSC: CD11b^+^CD33^+^CD15^-^ G-MDSC: CD11b^+^CD33^+^HLA-DR^low/-^CD14^-^ CD15^+^	G-MDSC are increased in BMMC of MM patients (highest in RRMM) VS. MGUS/SMM patients or HD (p < 0.05). G-MDSC in BMMC and PBMC of MM patients expressed higher levels of PD-L1 (p < 0.05).	(42)
		PB BM	G-MDSC: HLA-DR^low/-^CD33^+^CD11b^+^CD15^+^CD14	There is an association between high G-MDSC levels and poor OS in PB and BM of MM patients vs. HD (p < 0.05, p < 0.01).	(43)
		PB	M-MDSC: CD33^+^CD11b^+^HLA-DR^low/-^CD14^+^CD15 G-MDSC: CD33^+^CD11b^+^HLA^-^DR^low/-^CD14^-^ CD15^+^	The G-MDSC subpopulation was increased in samples from patients with MM (both patients with progressive disease and patients with stable disease vs. age-matched controls, p < 0.0001, p < 0.0445.)	(44)
		PB	M-MDSC: CD66b^+^CD15^-^CD14^+^HLA-DR^-^ G-MDSC: CD66b^+^CD15^+^CD14^-^HLA-DR^-^	G-MDSC and M-MDSC were increased in PB of MM VS. HD (p < 0.0001). Argl^+^G-MDSC percentage was increased in PB of ND MM patients VS. MGUS (p < 0.0001), and it was higher in RRMM VS. ND (p < 0.0001).	(45)
		BM	G-MDSC: CD11b^+^CD13^+^CD16^+^	G-MDSCs are defined as CD11b^+^CD13^+^CD16^+^ neutrophils in MM.	(46)

PB, peripheral blood; BM, bone marrow; HD, health donors; AML, acute myeloid leukemia; CML, chronic myeloid leukemia; B-ALL, B-Cell acute lymphoblastic leukemia; CLL, chronic lymphocytic leukemia; DLBCL, diffuse large B cell lymphoma; HL, Hodgkin’s lymphoma; B-NHL, B-Cell non-Hodgkin lymphoma; M-MDSC, monocytic myeloid derived suppressor cells; G-MDSC, granulocytic myeloid derived suppressor cells; IL-10, interleukin 10; TGF-β1, transforming growth factor beta 1; Th cells, helper T-cells; ND, newly diagnosed; Rel, relapsed; Rem, remission; IPI, international prognostic index; EFS, event-free survival; PFS, progression-free survival; BMMC, bone marrow mononuclear cell; PBMC, peripheral blood mononuclear cell; RRMM, relapsed/refractory multiple myeloma; OS, Overall survival; MGUS, monoclonal gammopathy of undetermined significance.

### 2.1 Leukemia

Acute Myeloid Leukemia (AML) represents the most common myeloid malignancy and is characterized by the expansion of immature myeloid progenitors or blasts in the BM and peripheral blood (PB) (51). In AML, distinct MDSC subsets have been characterized and specifically the circulating M-MDSC subset (defined as CD14^+^HLA-DR^low^) appeared to be elevated and correlated with a poor prognosis in AML patients (21, 22). In addition, eMDSC (CD33^+^CD11b^+^HLA-DR^−/Low^CD14^−^CD15^−^) were also increased in the PB of AML patients, however its impact of prognosis remains unknown (23). Interestingly, Sun et al. observed a correlation between the total number of MDSC in the BM (CD33^+^CD11b^+^HLA-DR^low/−^) and minimal residual disease (MRD) (determined by flow cytometry), as MDSC levels in the high MRD group (MRD > 1×10^−2^) was significantly higher than that in the middle (1x10^−2^ > MRD > 1×10^−4^) and the low (MRD < 1×10^−4^) MRD groups (24).

Chronic Myeloid Leukemia (CML) is a hematopoietic stem cell malignancy characterized by the acquisition of the t (9, 48) chromosomal translocation leading to expression of the BCR/ABL oncogene (52). Both M-MDSC (CD14^+^HLA-DR^−^) and G-MDSC (CD11b^+^CD33^+^CD14^-^HLA-DR^−^) were increased in the PB of CML patients compared to healthy controls and treatment with the tyrosine kinase inhibitor imatinib decreased the MDSC percentages to normal levels (25, 53). Although higher levels of G-MDSC could be detected in high-risk patients (based on Sokal score) compared to low-risk patients, its impact on prognosis needs to be further elucidated (26).

In precursor B cell Acute Lymphoblastic Leukemia (B-ALL), a malignancy of precursor B cells with the highest incidence among children, elevated levels of G-MDSC (CD45^+^CD19^−^HLA-DR^−^CD11b^+^CD33^+^CD15^+^) in the PB and BM of newly diagnosed patients has been observed (27). Similar to the findings in AML, a correlation could be observed between the G-MDSC levels, in the BM and PB, and MRD status of B-ALL patients at diagnosis. In addition, the frequency of G-MDSC correlated positively with other prognostic indicators including the percentage of CD20^+^ cells and blast cells (14, 27, 28).

Chronic Lymphocytic Leukemia (CLL) arises from the clonal expansion of CD5^+^ B lymphocytes in the BM (54). A study of 49 CLL patients demonstrated an upregulation of CD14^+^HLA-DR^low/−^ M-MDSC compared to healthy patients (29). In addition, the elevated levels of M-MDSC were significantly correlated with tumor progression and a poor prognosis of CLL patients (30). The negative impact of M-MDSC (CD14^+^CD11b^+^CD15^−^HLA-DR^−/low^) on the clinical outcome of CLL patients was also confirmed by Kowalska et al. (31). In contrast, the study by Ferrer et al. found a significant increase in the G-MDSC (HLA-DR^low^CD11b^+^CD33^+^CD15^+^) number of CLL patients which was associated with a poor clinical outcome. While CLL-derived G-MDSC suppressed T-cell growth *in vitro*, M-MDSC were less immunosuppressive due to the presence of TNFα and were defined as a more immunostimulatory subtype. The authors concluded that the G-MDSC appeared to be the preferred subtype to target, since they more effectively induce immune suppression in CLL patients (32).

### 2.2 Lymphoma

Diffuse large B cell lymphoma (DLBCL) is the most common type of non-Hodgkin lymphoma (NHL) hat develops from the B lymphocytes. Azzaoui et al. observed an increase in M-MDSC (CD14^+^HLA-DR^low^) and G-MDSC (Lin^-^HLA-DR^-^CD33^+^CD11b^+^) populations in DLBCL patients, however the M-MDSC were the only subset that could be correlated with the International Prognostic Index and event-free survival (33). This observation was confirmed by Wang et al. who demonstrated a significant increase in the circulating M-MDSC (CD14^+^CD33^+^HLA-DR^−/low^) of newly diagnosed and relapsed DLBCL patients and found that the level of M-MDSC could be used as a biomarker for poor prognosis of DLBCL patients (34).

The presence of Reed-Sternberg cells is a specific hallmark of Hodgkin lymphoma (HL). Romano et al. demonstrated that all circulating MDSC subsets (CD11b^+^CD33^+^CD14^−^CD34^+^HLA-DR^−^ or immature MDSC, CD11b^+^CD33^+^CD14^−^HLA-DR^−^ or G-MDSC, CD14^+^HLA-DR^low/−^ or M-MDSC) were increased in HL patients compared to normal subjects. Higher MDSC percentages were present in non-responders and CD34^+^ immature MDSC were predictive for a short progression-free survival in HL patients (35).

More recently, a study in B-NHL patients including CLL, DLBCL, marginal zone lymphoma (MZL), high‐grade B‐cell lymphoma (HGBL), mantle‐cell lymphoma (MCL), primary central nervous system lymphoma (PCNSL) and follicular lymphoma (FL) patients was carried out to investigate the impact of MDSC number and subsets (CD14^+^CD33^+^HLA‐DR^−/low^ for M‐MDSC, CD10^‐^HLA‐DR^−/low^ for G‐MDSC) on B-NHL patient’s prognosis. A significant increase could be observed in the levels of M-MDSC and G-MDSC in the diverse types of B-NHL compared to healthy donors. MDSC levels were closely associated with disease progression (tumor stage, LDH levels) and both subsets were defined as effective indicators of poor prognosis in B-NHL patients (36, 55).

### 2.3 Multiple myeloma

Multiple myeloma (MM) is a plasma cell malignancy in which monoclonal plasma cells proliferate in the BM (56). Controversial results were reported regarding the MDSC levels and subtypes present in MM patients. One of the first studies demonstrated elevated levels of M-MDSC (CD14^+^HLA-DR^−/low^) in MM patients at diagnosis compared to healthy controls (57). In addition, M-MDSC levels were correlated with tumor progression and MDSC levels could be considered as an indicator for the efficacy of therapy (37, 38). A study by Bae et al. recently confirmed the independent adverse prognostic impact of PB derived M-MDSC in patients with MM and suggested the analysis of M-MDSC as a prognostic marker in clinical practice (39). In the context of autologous stem cell transplantation (ASCT), lower M-MDSC levels were associated with a longer time to progression. Interestingly, pre-ASCT derived M-MDSC strongly inhibited the *in vitro* cytotoxic effect of melphalan; which could be reduced by the blockade of colony-stimulating factor 1 receptor (CSF1R) (40). However, more recent studies demonstrate a significant increase of G-MDSC (CD11b^+^CD33^+^HLA‐DR^−/low^CD14^-^CD15^+^) in BM and PB of MM patients compared to monoclonal gammopathy of undetermined significance (MGUS), smoldering MM patients and healthy controls, while no significance could be observed for M-MDSC (41–44). The increase in G-MDSC was also associated with MM disease activity and could be used to predict the response to immunomodulatory agent lenalidomide (45). Perez et al. also observed a correlation between the clinical significance, immunosuppressive potential, and transcriptional network of well-defined neutrophil subsets. In addition, they suggested a set of optimal markers (CD11b/CD13/CD16) for accurate monitoring of G-MDSC in MM patients (46).

## 3 Therapeutic approaches to target MDSC in hematological cancers

**Figure 1:**
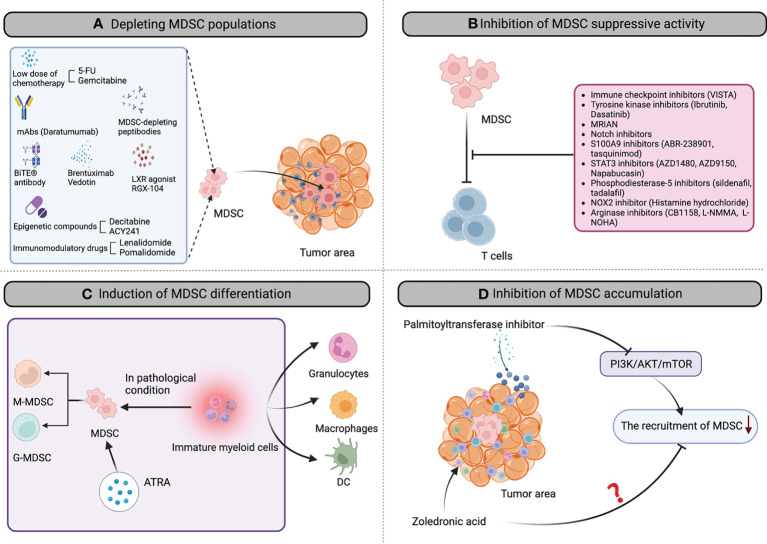
The landscape of MDSC-targeting strategies in hematological cancers. Multiple MDSC-targeting approaches were evaluated in hematological cancers to restore the anti-tumor immune response, including: **(A)** depleting MDSC populations through low-dose chemotherapy agents, mAbs, peptibodies, brentuximab vedotin, epigenetic compounds, CD33/CD3-bispecific T-cell engaging (BiTE^®^) antibody, LXR agonist RGX-104, Immunomodulatory drugs et al; **(B)** attenuating the immunosuppressive mechanisms of MDSC by immune checkpoint inhibitors, tyrosine kinase inhibitors, MRIAN, notch inhibitors, S100A9 inhibitors, STAT3 inhibitors, phosphodiesterase-5 inhibitors, histamine hydrochloride; arginase inhibitors; **(C)** inducing the differentiation of MDSC into mature myeloid cells by all-trans-retinoic acid (ATRA) to reduce MDSC population and remove their immunosuppression; **(D)** inhibiting MDSC accumulation in the tumor microenvironment by palmitoyltransferase inhibitor and zoledronic acid. mAb, monoclonal antibody; BiTE, bi-specific T-cell engagers; 5-FU, 5-fluorouracil; LXR, activation of liver X receptor; STAT3, signal transducer and activator of transcription 3; NOX2, NADPH oxidase 2; ATRA, all-trans-retinoic acid; DC, dendritic cell.

In past years, some specific and various unspecific strategies have been investigated to either modulate the MDSC suppressive function, affect their differentiation/maturation potential, block MDSC development or deplete this cell population in the tumor microenvironment. Below, and in **Figure 1** and **Table 2**, we will summarize all strategies that have been tested in the context of hematological cancers.

**Table 2:** Overview of MDSC-targeting approaches in hematological cancers.

Agents		Disease	Model	Mechanisms/ Functions	Ref
Cytotoxic therapies	5-FU	Lymphoma	EL-4 syngeneic model	Gemcitabine and 5-FU decreased the number of MDSC.	(58)
	Gemcitabine	MM Lymphoma	5T33MM model A20 syngeneic model E.G7-OVA model	Targeting MDSC by anti-GR1 antibodies and 5-FU reduced tumor load. Accumulation of MDSC in the spleen of lymphoma-bearing mice. Lipid nanocapsules loaded with a lauroyl-modified form of gemcitabine efficiently target the M-MDSC subset.	(59) (60, 61)
Monoclonal antibodies	Daratumumab	MM	Patient PB, BM samples	G-MDSC expressed elevated CD38 and were highly sensitive to daratumumab-mediated ADCC/CDC. Daratumumab-mediated depletion of M-MDSC using a combination of daratumumab and cetrelimab in RRMM patients.	(62) (63)
MDSC-depleting peptibodies	Peptibodies	Lymphoma	EL-4 syngeneic model	*In vivo*, intravenous peptibodies injection depleted blood, splenic and intra- tumoral MDSC. S100 family proteins were identified as candidate targets.	(64)
Brentuximab Vedotin		HL	Patient PB samples	BV reduced the absolute number of three MDSC subtypes and s-Arg-1 levels. Patients with baseline s-Arg-1 >200 ng/ml had inferior PFS at 36 months.	(65)
Epigenetic compounds	Decitabine	Lymphoma Leukemia MM	EL-4 syngeneic model WEHI-3 model MPC-11 model	DAC treatment depleted MDSC *in vivo*. DAC activated adaptive T-cell response *in vitro* and autologous T-cell response to tumor cells *in vivo* by depleting MDSC.	(66)
				DAC treatment inhibited MPC-11 proliferation *in vivo* by depleting M-MDSC and increasing T-cell infiltration in tumor tissue.	(67)
	ACY241	MM	Patient BM samples	ACY241 decreases the frequency and expression of immune checkpoints on CD138^+^ MM cells, regulatory T-cells and MDSC.	(68)
CD33/CD3-bispecific BITE® antibody	AMG330	Leukemia	Primary AML-blasts	AMG330 triggers T-cell mediated lysis of AML-blasts that is further enhanced by MDSC depletion.	(69)
	AMV564	MDS	MDS BM primary samples, CD33hi SKM1 xenograft model	AMV 564 showed anti-tumor activity by immunodepletion of MDSC in primary MDS patients and in a disseminated leukemia mouse model.	(70)
LXR agonist RGX- 104	RGX-104	Lymphoma		LXR agonist treatment promotes MDSC apoptosis *in vitro* and *in vivo*. Patient blood sample analysis revealed a depletion of G-MDSC after treatment of cancer patients with RGX-104.	(71)
Immunomodulatory drugs	Lenalidomide Pomalidomide	MM	Patient PB, BM samples	LEN and POM prevent MDSC induction through transcriptional expression and production of CCL5 and MIF, and increased the mRNA level of IRF8 (a negative regulator of differentiation towards MDSC) in PBMC.	(72)
Immune checkpoint inhibitors	VISTA- targeting	AML	Patient PB samples C1498 syngeneic PD-1H knockout model	VISTA is highly expressed on MDSC in patients, and increased in ND patients. VISTA knockout/targeting diminished the inhibition of CD8 T-cell activity by MDSC in AML. VISTA on host cells and AML cells induces immune evasion in AML.	(73, 74)
Tyrosine kinase inhibitors	Ibrutinib	CLL	A cohort of previously untreated CLL patients, PBMC samples	Ibrutinib therapy selectively alters the numbers of MDSC, CD4^+^ and CD8^+^ T-cells and Th-cell subsets *in vivo*.	(32)
	Dasatinib	CML	Patients and age-matched HD PB samples	The percentage of M-MDSC correlates with MMR in patients treated with dasatinib.	(75, 76)
Metabolic Reprogramming Immunosurveillance Activation Nanomedicine	MRIAN	T-ALL	Activated Notchl mutant driven T-ALL model	MRIAN efficiently penetrates BM and selectively targets leukemic cells and MDSC in T-ALL mice. MRIAN Inhibits mitochondrial metabolism and reduces ROS levels in MDSC.	(77)
Notch inhibitors	ADAM10 Anti-Jagged antibody	T-ALL Lymphoma	ADAM10 transgenic (A10Tg) model Patient PB samples Notch3-transgenic T-ALL model Notchl-activated KE-37 cell line and HD PB EL-4 syngeneic model	ADAM10 overexpression in transgenic mice resulted in a systemic expansion of MDSC. The accumulation of MDSC was attributed to the differential cleavage of Notch in S2 and S3 products by ADAM10. Daratumumab-mediated depletion of M-MDSC using a combination of daratumumab and cetrelimab in RRMM patients Notch-signaling deregulation in immature T-cells promotes CD11b^+^Grl^+^ MDSC in the Notch3-transgenic murine model of T-ALL. Human Notch-Dependent T-ALL cell lines induce MDSC from HD PBMC. Tumors induce Jagged ligands in MDSC through NFκB-p65. Anti-Jagged therapy induces an anti-tumor effect, and impacts the suppressive activity of tumor-MDSC.	(78–80)
S100A9 inhibitors	ABR-238901	MM	5T33MM model	Blocking S100A9 interactions with ABR-238901 did not directly affect MDSC accumulation but did reduce IL-6 and IL-10 expression by MDSC. ABR-238901 treatment in combination with bortezomib resulted in an increased reduction in tumor load compared with single treatments.	(81)
	Tasquinimod		5T33MM model 5TGM1 model	Tasquinimod has direct anti-tumor effects *in vivo*. Tasquinimod targets M-MDSC and increases serum interferon-gamma.	(82)
STAT3 inhibitors	AZD9150	NHL (primarily DLBCL)	Patient PB	AZD9150 therapy resulted in a decrease of G-MDSC and increased CD4 and CD8 T-cells in three out of four NHL patients.	(83)
Phosphodiesterase-5 inhibitors	Sildenafil	B cell lymphoma	A20 syngeneic model	IL-4Ra expression on MDSC correlates with tumor progression and can be inhibited by sildenafil.	(84)
	Tadalafil	MM	Case report MM patient	Tadalafil, in a patient with end-stage RRMM reduced MDSC function and generated a dramatic and durable anti-myeloma immune and clinical response.	(85)
			Clinical trial of MM patients (refractory to lenalidomide-based regimens	MDSC were not detected in any of the patients at baseline in both blood and BM. No clinical response could be observed.	(86)
NOX2 inhibitor	Histamine hydrochloride	Lymphoma	EL-4 syngeneic model	HDC reduces tumor progression by targeting NOX2^+^ MDSC. HDC significantly reduced the accumulation of MDSC within EL-4 lymphomas.	(87)
Arginase inhibitor	nor-NOHA CD1158	MM	Patient PB samples	T-cell proliferation and cell cytotoxicity is enhanced by PMN-SN in the presence of arginase inhibition. T-cell cytokine secretion is hyperactivated by PMN-SN in the presence of arginase inhibition.	(16, 88)
		AML	AML mice NOG-SCID mice	The AML mice had significant reductions in plasma arginine compared to controls. The arginine depleting therapy can inhibit antigen-dependent T cell responses *in vitro* and *in vivo*.	(89)
All-trans-retinoic acid		Lymphoma	EL-4 Syngeneic model	ATRA induces expression of GSS and accumulation of GSH in MDSC.	(90)
		APL	Transgenic PML-RARA APL model T-cell depletion in APL B6 model HIS APL model	In PML-RARA mice, the remission following ATRA treatment was accompanied with normalized levels of PGD2, ILC2s, M-MDSC, and a recovery of activated CD8+ T-cells. T-cell depleted APL B6 mice showed a shorter survival and an increase in ILC2 and M-MDSC. The increase in PGD2 and a major accumulation of ILC2 and M-MDSC upon leukemia engraftment were observed in HIS APL mice that were reverted by ATRA therapy.	(91)
PalmitoyItransferase inhibitor	2-BP	AML	Patient PB samples	Palmitoylated proteins on the AML-EVs' surface contribute to the TLR2-dependent MDSC reprogramming	(92)

5-FU, 5-fluorouracil; BV, brentuximab vedotin; DAC, decitabine; LEN, lenalidomide; POM, pomalidomide; VISTA/PD-1H, v-domain immunoglobulin suppressor of T-cell activation; MRIAN, metabolic reprogramming immunosurveillance activation nanomedicine; S100A9, calgranulin B or myeloid-related protein 14, MRP14; LXR, activation of liver X receptor; ADAM10, a disintegrin and metalloprotease 10; HDC, histamine hydrochloride; PMN-SN, polymorphonuclear neutrophil granulocytes supernatants; ATRA, all-trans-retinoic acid; MDS, myelodysplastic syndromes; T-ALL, T‐cell acute lymphoblastic leukemia; APL, acute promyelocytic leukemia; HIS, humanized mice; ADCC/CDC, Fc-mediated antibody-dependent cellular cytotoxicity and complement-dependent cytotoxicity; CCL5, C-C Motif Chemokine Ligand 5; MIF, macrophage migration inhibitory factor; IFR8, interferon regulatory factor 8; ROS, reactive oxygen species; NFκB-p65, nuclear factor kappa-light-chain-enhancer of activated B cells; IL6, interleukin 6; CTLs, cytotoxic T lymphocyte; GSS, glutathione synthase; GSH, glutathione; PDG2, a receptor for prostaglandin D2; ILC2, group 2 innate lymphoid cells.

### 3.1 MDSC depleting agents

#### 3.1.1 Cytotoxic therapies

5-Fluorouracil and Gemcitabine, both chemotherapeutic compounds routinely used in the clinic for the treatment of cancer, have been described to decrease the number of MDSC in preclinical mouse models of hematological cancers (58–60). Due to the low selectivity and dose-dependent toxicity, various encapsulated gemcitabine formulations have been developed and examined for safety and tumor-directed toxicity. Sasso et al. demonstrated that low dose gemcitabine-loaded lipid nanocapsules efficiently targeted the M-MDSC subset and relieved tumor-associated immunosuppression *in vitro* and *in vivo* using the E.G7-OVA lymphoma model. The efficient uptake of the nanocapsules into the M-MDSC subset was attributed to a mechanism called ‘macropinocytosis’. Moreover, authors found that preconditioning with low dose gemcitabine-loaded lipid nanocapsules enhanced the efficacy of adoptive T-cell therapy in the E.G7-OVA tumor model, further illustrating its potential as immune modulating therapy in cancer (61).

#### 3.1.2 Monoclonal antibodies

Daratumumab is an anti-CD38 monoclonal antibody, FDA approved in 2015 for the treatment of relapsed/refractory MM patients. Besides the ubiquitous expression of CD38 on MM cells, CD38 antigen is also expressed by other cell types including MDSC and regulatory B cells (62). Krejcik et al. demonstrated that *in vitro* generated G-MDSC (CD11b^+^CD14^–^HLA–DR^–^CD15^+^CD33^+^) expressed elevated CD38 and were highly sensitive to daratumumab-mediated ADCC/CDC compared with the isotype control. Findings were confirmed in patients treated with a combination of lenalidomide, dexamethasone with or without daratumumab. Using western blot analysis, a selective reduction of M-MDSC was observed in patients treated with the triple combination compared to patients treated with dexamethasone and lenalidomide (93). Data obtained by Cohen et al. further supported the daratumumab-mediated depletion of M-MDSC using a combination of daratumumab and anti-PD-1 monoclonal antibody cetrelimab in relapsed/refractory MM patients (63).

#### 3.1.3 MDSC-depleting peptibodies

Using a competitive peptide phage display platform, candidate peptides were identified that specifically bind to MDSC derived from EL4 mice, a murine lymphoblastic tumor model. Peptides were fused with the Fc portion of mouse IgG2b to generate MDSC-specific peptibodies. *In vivo* studies in lymphoma models including A20, EL4 and E.G7-OVA demonstrated that the peptibodies were able to deplete intra-tumoral MDSC, without affecting other inflammatory cell types (e.g., dendritic cells and T-cell). In contrast to anti-GR1 depleting antibodies which preferentially eliminate G-MDSC, peptibodies were able to deplete both M-MDSC and G-MDSC subsets. Peptibodies significantly delayed tumor growth in EL4 mice and alarmins S100A8/S100A9 were identified as potential candidate targets expressed by the MDSC (64, 94).

#### 3.1.4 Brentuximab vedotin

Brentuximab Vedotin (BV) is an antibody-drug conjugate designed to selectively deliver monomethylauristatin E, a microtubule-disrupting agent, to CD30-expressing cells. The compound has been FDA approved in 2018 for the treatment of patients with previously untreated stage III or IV classical HL in combination with chemotherapy (95). Although it remains unclear whether CD30 is expressed or not on MDSC subsets, Romano et al. demonstrated that BV reduced the absolute number of three MDSC subtypes (CD11b^+^CD33^+^CD14^−^CD34^+^HLA-DR^−^; M-MDSC and G-MDSC) coinciding with reduced soluble Arg-1 levels and restored the entire T-cell populations in HL patients; indicating its therapeutic use as MDSC targeting agent (65).

#### 3.1.5 Epigenetic compounds

5-aza-2′-deoxycytidine, also known as decitabine (DAC), has been shown to act as an irreversible inhibitor of DNA methyltransferases and induces gene-specific DNA demethylation when administered at a low dose (96). Besides the reactivation of tumor suppressor genes through demethylation, DAC exerts pleiotropic effects on the tumor immune microenvironment including the upregulation of MHC-I/MHC-II expression levels, the increased expression of co-stimulatory molecules and the targeting of immunosuppressive cell types. The effect of DAC on MDSC subtypes was analyzed in leukemia (WEHI-3), lymphoma (EL4) and MM (MPC11) models *in vitro* and *in vivo*. DAC treatment induced MDSC apoptosis (CD11b^+^GR1^+^) *in vitro* and increased T-cell activation in leukemia and lymphoma models. In the MCP11 MM model, DAC inhibited MM cell proliferation and induced an autologous T-cell immune response by depleting the M-MDSC subset in the MM BM microenvironment (66, 67).

Histone deacetylase (HDAC) inhibitors (e.g. entinostat, valproic acid, vorinostat) are another class of epigenetic compounds and were also reported to reduce MDSC levels or inhibit MDSC suppressive capacity in solid tumor models (97). Treatment of BM mononuclear cells of MM patients with ACY241, an HDAC6 selective inhibitor, significantly reduced the HLA-DR^low/-^CD11b^+^CD33^+^ MDSC population, while it augments the immune response as evidenced by increased perforin/CD107a expression, IFN-γ/IL-2/TNF-α production and antigen-specific central memory cytotoxic T lymphocytes (68).

#### 3.1.6 CD33/CD3-bispecific T-cell engaging (BiTE^®^) antibody

AMG 330 is the first BiTE^®^ developed against CD33, an antigen that is not only expressed on the majority of AML-blasts, but also on M-MDSC (98). Jitschin et al. observed an increase in the percentage of HLA-DR^low^ (CD14^+^CD11b^+^) M-MDSC, that co-express CD33, in newly diagnosed AML patients compared to healthy controls. In the presence of AMG 330, T-cell were able to eliminate CD33^+^IDO^+^ *in vitro* generated MDSC. Adding MDSC to co-cultures of T-cell and AML cells resulted in reduced AML-blast killing, while the addition of an IDO inhibitor promoted the AMG 330-mediated clearance of AML-blasts. Data suggest a dual anti-tumor effect of AMG 330 through increased T-cell mediated cytotoxicity against AML blasts and CD33^+^ MDSC (69). Another study by Cheng et al. evaluated the effects of AMV 564, a novel bivalent CD33/CD3 T-cell engager and showed immunodepletion of MDSC and anti-tumor activity using primary samples of myelodysplastic syndrome (MDS) patients and a disseminated leukemia mouse model (70).

#### 3.1.7 LXR agonist RGX-104

Liver-X nuclear receptors (LXR) are members of the nuclear hormone receptor family that drive, among others, the transcriptional activation of ApoE. Masoud et al. observed that an LXR agonist RGX-104 induces apoptosis of MDSC and enhances T-cell activation in solid tumor models (71). RGX-104 is currently evaluated in an ongoing phase 1 clinical trial in patients with metastatic solid cancers or lymphomas that have progressed on standard therapies (NTC02922764). Blood sample analysis revealed a depletion of G-MDSC and increased T-cell activation after treatment of cancer patients with RGX-104.

#### 3.1.8 Immunomodulatory drugs

Immunomodulatory drugs (IMiDs), including lenalidomide and pomalidomide, are a group of drugs that are derivatives from thalidomide and are routinely used in the treatment of MM (99). Kuwahara-Ota et al. examined the impact of IMiDs on MDSC *in vitro* and found a significant reduction in MDSC level upon coculture of MM-derived PB mononuclear cells and human MM cell lines, with pomalidomide being more potent than lenalidomide (72). However, clinical evidence supporting this hypothesis is missing as lenalidomide-treated patients showed a higher abundance of CD14^+^CD15^+^ MDSC. Moreover, *in vitro* findings by Görgun et al. demonstrated that lenalidomide could not overcome MDSC-mediated T-cell suppression in MM (100). In the A20 lymphoma tumor model, a lenalidomide-associated reduction in systemic MDSC number and increased immune activation has been observed, further illustrating the controversy regarding the impact of lenalidomide on MDSC populations, depending on the used tumor model and type.

### 3.2 Inhibition of MDSC suppressive activity

#### 3.2.1 Immune checkpoint inhibitors

In MM, the immune checkpoint PD-L1 was significantly higher expressed on the G-MDSC subset of BM and PB-derived MM patients (newly diagnosed and relapsed) compared to G-MDSC of MGUS and healthy individuals (42). Although some studies in solid tumors suggest that PD-L1 blocking could partially restore the MDSC suppressive function, Ahn and colleagues could not observe any effect of a PD-L1 blocking antibody on splenic MDSC number or subsets in the MOPC-315 immunocompetent MM model (101–104). To fully elucidate whether PD-L1 expression on MDSC is linked to its suppressive function, additional studies are required in MM models that allow BM-derived MDSC investigation as well.

V-domain Ig suppressor of T-cell activation (VISTA) or PD-1H is a novel checkpoint regulator that is predominantly expressed in the hematopoietic compartment, and particularly within the myeloid lineage (105). In solid tumors, inhibition of VISTA resulted in improved anti-tumor immune responses *in vivo* and currently clinical trials are ongoing to assess its therapeutic potential in advanced solid tumor malignancies (NCT05082610, NCT04475523) (106). In AML, VISTA was found to be highly expressed on monocytes (CD45^int^CD11b^+^CD14^high/low^) and myeloid leukemia blasts (CD45^in^ vs. SCC). VISTA expression on PB-derived MDSC (CD11b^+^CD33^+^ HLA-DR^−^) was significantly higher in AML patients compared to healthy controls. In addition, siRNA mediated knockdown of VISTA in MDSC resulted in increased T-cell proliferation *in vitro* and diminished the MDSC-mediated suppression of CD8^+^ T-cell. Strikingly, the authors observed a strong correlation between VISTA-expressing MDSC and PD-1 expressing T-cells (including CD4, CD8 and Treg), indicating a link between both checkpoints to suppress the immune system in AML patients (73). In another study, VISTA-expressing murine myeloid leukemia cells were injected into wild type and PD-1H (VISTA) knock out mice. Authors observed a reduction in AML cell growth in PD-1H knock out mice, which was further diminished by the administration of PD-1H blocking antibodies. These data suggest that VISTA expression on both the host cells and AML cells are involved in the cancer immune evasion. Moreover, epigenetic modulation using DAC further increased the overall survival of PD-1H knock out mice, indicating the potential of combining both compounds in clinical setting (74).

#### 3.2.2 Tyrosine kinase inhibitors

Ibrutinib is a first-in-class oral irreversible inhibitor of Bruton Tyrosine Kinase (BTK), a critical enzyme in the B-cell receptor signaling cascade, and is highly effective in the treatment of CLL, MCL and Waldenstrom’s macroglobulinaemia. BTK has been described to be expressed by MDSC and treatment with ibrutinib was found to affect the MDSC generation and function in solid tumor models, indicating its therapeutic potential to increase immune-based therapies (107, 108). A study by Ferrer et al. demonstrated that G-MDSC were the preferential subset to target in CLL patients to increase T-cell function. Three months ibrutinib therapy of CLL patients resulted in a significant decline of G-MDSC, while M-MDSC and monocytes remained unaffected. While ibrutinib had no direct effect on the T-cell suppressive activity, it skewed the T-cell differentiation to T helper 1 cells in the presence of MDSC, indicating a change from an immunosuppressive towards a more immune effective state (32).

The effect of other tyrosine kinase inhibitors including imatinib, nilotinib and dasatinib on MDSC levels was evaluated in CML patients. All compounds induced a significant reduction in G‐MDSC at 3–6 months and 9–12 months of treatment. However, the M-MDSC subset was not significantly changed during imatinib and nilotinib therapy and was only reduced in dasatinib‐treated patients. Interestingly, a significant correlation was found between the major molecular response (MMR) values and number of persistent M‐MDSC at 12 months of dasatinib treatment, indicating its prognostic value in these patients (75, 76).

#### 3.2.3 Metabolic modifier MRIAN

Metabolic Reprogramming Immunosurveillance Activation Nanomedicine (MRIAN) is an L-phenylalanine polymer, developed to target the immunosuppressive BM microenvironment by inhibiting MDSC. MRIAN reduced ROS levels and induced MDSC differentiation towards functional immune cells (e.g., macrophages, natural killer cells, dendritic cells). In T-ALL mice, MRIAN significantly improved the T-cell number and function by inhibiting MDSC. Studies also demonstrated an enhanced cellular uptake of MRIAN in T-ALL cells and MDSC compared to normal hematopoietic cells and progenitors. MRIAN assembled to doxorubicin (MRIAN-Dox) demonstrated an enhanced anti-tumor efficacy and reduced toxicity profile (including cardiotoxicity and myeloablation side effects) in T-ALL mice; indicating its therapeutic potential as metabolic modifier to target MDSC (77).

#### 3.2.4 Notch inhibitors

The Notch signaling pathway has been identified to play a key role in MDSC accumulation (109–111). In transgenic mice overexpressing ADAM10, a Notch processing enzyme, an accumulation of systemic CD11b^+^Gr1^+^ MDSC was found (78). A study by Grazioli et al. observed an expansion of MDSC in a transgenic mouse model of Notch3-dependent T-ALL. Interestingly, using both *in vitro* and *in vivo* experiments, they found that CD4^+^CD8^+^ T-cell (derived from the Notch3-transgenic mice) were the drivers of MDSC accumulation, through a mechanism that was dependent on both Notch and IL6. Conversely, anti-Gr1-mediated depletion of MDSCs in T-ALL-bearing mice significantly reduced the proliferation and expansion of malignant T-cell. These data were confirmed by coculturing human Notch-dependent T-ALL cell lines and healthy donor derived PB mononuclear cells *in vitro*, resulting in increased CD14^+^HLA-DR^low/neg^ MDSC accumulation and T-cell suppression; effects that were not observed with T-ALL cells that did not express Notch1- or Notch3-activated protein (79).

Another therapeutic approach to alter Notch signaling is the use of anti-Jagged blocking antibodies. Sierra et al. assessed the anti-tumor and immunogenic effect of CTX014, a humanized IgG1 blocking antibody, cross-reactive for both mouse and human Jagged1 and 2, in solid and hematological tumor models. Surprisingly, results demonstrated an increase of CD11b^+^GR1^+^ MDSC in tumors of mice treated with anti-Jagged therapy compared to vehicle. Data suggested that anti-Jagged therapy triggered an anti-tumor immune response through induction of immunogenic MDSC-like cells. Anti-tumor and immunogenic effects of anti-Jagged therapy was evaluated in an E.G7-OVA T-cell lymphoma model in combination with adoptive T-cell transfer of OT-I cells. Results showed that anti-Jagged therapy could overcome tumor-induced immune tolerance and increased the effect of the T-cell based immunotherapy (80).

In solid tumors, targeting Notch using γ-secretase inhibitors significantly increased the MDSC number in preclinical cancer models (112). There was a specific increase in the G-MDSC subset and a downregulation of CD80, CD115 and CD124 markers, all associated with MDSC suppressive function. Using short hairpin constructs against *RBP-J*, Notch signaling was attenuated in BM cells and this resulted in reduced MDSC suppressive capacity. In addition, injection of RBP-J-deficient MDSC in tumor-bearing mice significantly reduced the tumor growth compared to controls (79, 113).

Altogether, these studies revealed a role of Notch signaling in the accumulation and suppressive function of MDSC in tumor-bearing mice. However, whether the effect is direct, indirect or a combination of both remains to be elucidated.

#### 3.2.5 S100A9 inhibitors

S100A9 is a calcium-binding protein, mainly secreted by granulocytes and monocytes, and has been reported to be essential for MDSC survival and accumulation in tumor-bearing mice including MM and lymphoma models (114). In MM, our group demonstrated the expression of S100A9 and its receptor TLR4 in both monocytic and granulocytic MDSC subsets. S100A9 acted as a chemoattractant for MM cells *in vitro* and induced the expression of pro-inflammatory cytokines by MDSC (e.g., TNFα, IL-6, IL-10). Targeting the interaction of S100A9 and its receptors using ABR-238901 did not affect MDSC accumulation, but significantly reduced cytokine expression by MDSC. Moreover, anti-angiogenic and anti-MM effects were observed *in vivo* using a combination therapy of ABR-238901 and bortezomib (81). Recently, we also investigated the effects of S100A9 inhibitor tasquinimod, currently evaluated in clinical trial for relapsed/refractory MM patients and observed a clear reduction in the M-MDSC subset *in vivo* (NCT04405167). In addition, tasquinimod abolished the immunosuppressive activity of *in vitro* generated MDSC, illustrating its potential as an immunotherapeutic compound (82).

#### 3.2.6 STAT3 inhibitors

Although STAT3 activation is known to play a pivotal role in MDSC accumulation and function, the effects of STAT3 inhibitors on MDSC activity is rather controversial (115, 116). AZD1480, a small-molecule inhibitor of JAK1/2 kinase, significantly decreased MDSC number and delayed tumor growth in MO4 melanoma-bearing mice. Despite a decrease in MDSC percentage, Maenhout et al. observed an enhanced MDSC-suppressive capacity and impaired T-cell proliferation and IFN-γ secretion upon treatment with AZD1480 (117). AZD9150, a next-generation antisense oligonucleotide inhibitor of STAT3, also demonstrated potent anti-tumor effects of lymphoma cell lines and in preclinical lymphoma models (83). The inhibitor was evaluated in a small group of non-HL patients and three out of four patients showed a decrease in the circulating G-MDSC population and an increase in CD4^+^ and CD8^+^ T-cell (118). Napabucasin, another STAT3 inhibitor, was also found to abrogate the MDSC suppressive function in solid tumors and exhibited potent cytotoxicity against NHL cell lines (119, 120). However, napabucasin-mediated MDSC-targeting and modulation has not been investigated in hematological cancers so far.

#### 3.2.7 Phosphodiesterase-5 inhibitors

Phosphodiesterase-5 (PDE5) inhibitors (e.g., sildenafil, tadalafil, vardenafil), particularly used for nonmalignant conditions in the clinic, have been found to increase anti-tumor immune responses by altering the MDSC suppressive function and restoring anti-tumor immunity (121). Using the A20 lymphoma model, it has been found that IL4Rα expression on MDSC correlated with tumor progression and could be inhibited using sildenafil. In addition, sildenafil reduced lymphoma-induced T-cell anergy and expansion of regulatory Treg (84). A case report of a patient with end-stage relapsed/refractory MM showed that the addition of tadalafil to its treatment regimen (lenalidomide, clarithromycin, dexamethasone) reduced the MDSC suppressive activity, as illustrated by a reduction in IL4Rα^+^, iNOS, Arg-1 and ROS. Interestingly, the changes in MDSC function were more pronounced in the BM compared to the blood and were associated with an increase in T-cell function (↑ IFNγ expression). With the administration of tadalafil, the patient could tolerate the combination of lenalidomide and dexamethasone and achieved a very good partial response (+/- 90% reduction in tumor burden) (85). Although a clinical trial was initiated combining tadalafil, dexamethasone and lenalidomide in MM patients who were refractory to lenalidomide-based regimens, the study was terminated at an early stage due to a lack of response. The limited efficacy could be explained by the low number of MDSC present in the patients at the time of enrollment, potentially attributed to the pre-treatment with lenalidomide (86). Further studies are required to investigate the impact of PDE-5 inhibitors in patients with elevated MDSC levels.

#### 3.2.8 Histamine hydrochloride

Histamine hydrochloride (HDC) is a NOX2 inhibitor and is known to inhibit the immunosuppressive function of myeloid cells by reducing ROS production (122). Low-dose IL-2 combined with HDC is approved in Europe for remission maintenance in adult AML patients. Grauers et al. further unraveled the impact of HDC on MDSC number and function using the EL4 lymphoma tumor model. HDC significantly reduced MDSC number *in vivo* and altered the MDSC-induced immunosuppression of T-cells *ex vivo*. Moreover, using Nox2 knock out mice and GR1-depleting antibodies, it has been suggested that HDC exerted its anti-tumor effects by targeting the NOX2^+^ GR1^+^ cells *in vivo*. Finally, authors also observed an enhanced anti-tumor efficacy using the combination of HDC and anti-PD-1 antibodies in the EL4 lymphoma model. HDC-mediated effects on MDSC were further evaluated using blood samples of AML patients that received HDC in conjunction with low-dose IL-2 for relapse prevention (NCT01347996) (87). HDC/IL-2 therapy resulted in a significant reduction in the frequency and absolute counts of M-MDSC, and this strong reduction significantly predicted the leukemia-free survival.

#### 3.2.9 Arginase inhibitors

Arginase is a key enzyme involved in the immunosuppressive function of G-MDSC. Romano et al. demonstrated that Arg-1 is mainly expressed by G-MDSC in MM, and that both Arg-1 and G-MDSC are reduced after treatment with lenalidomide *in vivo* (45). Interestingly, Vonwirth et al. demonstrated that arginase inhibition, using nor-NOHA or CB-1158, could reduce T-cell anergy of MM patients in the presence of supernatant derived from polymorphonuclear neutrophil granulocytes (~G-MDSC). In preclinical solid tumor models, arginase inhibitor CB-1158 inhibited MDSC-mediated immunosuppression, increased T-cell proliferation and activity, and reduced tumor growth *in vivo* (89). A first-in-human phase 1 study in solid tumors demonstrated that CB-1158 was well tolerated and achieved on-target inhibition as illustrated by the increase in plasma arginine (16, 88). Moreover, arginase inhibition has been proposed as an interesting adjuvant therapy by Mussai et al. in leukemia patients. Inhibition of the arginine metabolism by L-NMMA and L-NOHA enhanced the proliferation and cytotoxicity of anti-NY-ESO (AML associated cancer-testis antigen) T-cells against epigenetically-treated AML blasts. In addition, it could also boost the anti-CD33 Chimeric Antigen Receptor T-cell cytotoxicity against AML, further illustrating its potential as adjunct therapy in hematological cancers (89).

### 3.3 Induction of MDSC differentiation

#### 3.3.1 All-trans-retinoic acid

All-trans-retinoic acid (ATRA), a vitamin A derivative, has been described as an inducer of myeloid cell differentiation and maturation, reducing MDSC number and inducing activation of immune responses in preclinical hematological and solid tumor models (90). In acute promyelocytic leukemia (APL) patients, peripheral ‘group 2 innate lymphoid cells’ (ILC2s) were found to be increased and hyperactivated, and in turn activated M-MDSC (CD14^+^CD33^+^) through IL-13 secretion. Using patient samples and APL mice, authors demonstrated that ATRA-treatment reversed the increase in ILC2 induced M-MDSC, accompanied by an increase in T-cell function *in vitro* and *in vivo* (91).

Unfortunately, due to a poor solubility and fast drug metabolism, the clinical application of ATRA has been limited. Recently, a drug encapsulated liposome formulation L-ATRA has been developed with sustained release properties. *In vitro* treatment of myeloid leukemia cell lines HL-60 and NB4 resulted in increased expression of myeloid differentiation markers CD11b and CD11c, illustrating its therapeutic potential to target MDSC (123).

### 3.4 Inhibition of MDSC accumulation

#### 3.4.1 Palmitoyltransferase inhibitor

It has been shown that CD14^+^HLA-DR^low^ M-MDSC accumulate in newly diagnosed AML patients. Tohumeken et al. found that AML-derived extracellular vesicles were taken up by conventional monocytes *in vitro* which subsequently underwent MDSC differentiation. Apparently, the presence of palmitoylated proteins on the surface of AML-derived extracellular vesicles was responsible for the activation of TLR2/Akt/mTOR signaling and accumulation of MDSC. TLR2 neutralizing antibodies, mTOR inhibitor rapamycin or palmitoyltransferases inhibitor 2-BP abolished the generation of MDSC, indicating its potential therapeutic application as MDSC-targeted therapies (92).

#### 3.4.2 Zoledronic acid

Zoledronic acid is a bisphosphonate used for the treatment of MM associated hypercalcemia and bone metastasis in solid tumors (124). Although no information is available on the MDSC-targeting potential of zoledronic acid in hematological malignancies, Porembka et al. observed a reduced MDSC accumulation and improved anti-tumor immune response in pancreatic cancer models (125). These data suggest that zoledronic acid might exert a dual role as anti-MM therapy, impacting on the bone disease and the accumulation of immunosuppressive cell types.

## 4 Other MDSC-targeting approaches tested in solid tumors

Although not yet tested in hematological malignancies, other specific/unspecific MDSC-targeting approaches might be considered in the future. For example, an indoleamine-pyrrole 2,3-dioxygenase (IDO) peptide vaccine has been developed and significantly decreased IDO-expressing MDSC. The peptide vaccine delayed tumor progression in solid tumors inoculated with either IDO^+^ or IDO^-^ tumor cells, indicating the therapeutic effect was partially mediated by targeting of the immunosuppressive environment (126).

Consistent with the results obtained using AMG 330 and AMV 564, CD33-directed therapy with gemtuzumab ozogamicin demonstrated MDSC depleting capacity in solid tumor models. CD33 was expressed on blood and tissue-derived MDSC of patients across different cancer subtypes, indicating its broad therapeutic potential (127).

## 5 Combinatorial approaches

The past years, immune checkpoint inhibitors and chimeric antigen receptor (CAR) T-cell therapies emerged as concomitant approaches to treat hematological cancers. However, the presence of immunosuppressive MDSC influences their efficacy. A study in large B-cell lymphoma patients receiving axicabtagene ciloleucel (axi-cel), a CD19-directed CART-cell therapy, demonstrated a clear association between poor CART-cell expansion and PB M-MDSC (128). Combinatorial approaches using CART- therapy or immune checkpoint inhibitors with MDSC-targeting agents (e.g., ATRA, gemtuzumab ozogamicin, AMV 564) clearly enhanced the anti-tumor efficacy in solid tumor models (127–129). These results imply the importance of using a similar approach in the treatment of hematological cancers.

## 6 Conclusion

Despite the controversy surrounding the nature and uniqueness of MDSC, there is no exists about their value as a therapeutic target in hematological cancers. MDSC contribute to tumor cell survival, immunosuppression and drug resistance; however, strategies to specifically eliminate this cell population or block their development are rather limited. Differences in analysis, tumor models, disease stages and treatment-related changes certainly contributed to the complexity to identify unique markers and specific approaches to tackle this cell type and reverse their immunosuppressive capacity. Further developments and applications of single-cell multi-omics will provide unique insights about the MDSC phenotypical markers and subsets, hopefully leading to a more specific MDSC-targeting approach in future. In addition, as MDSC are key regulators of immunosuppression, they contribute to the reduced effectiveness of current immunotherapeutic approaches including CAR-T therapy and immune checkpoint inhibitors. Specific targeting of these cell types in combination with other immunotherapies should be evaluated in clinical trials as this approach might be the key to increase anti-tumor immune responses and improve patient’s outcome.

## Author contributions

RF and KDV developed the design and arguments for the paper, drafted the manuscript and designed the figures. AM, NB, EM, EB, KV, KM, and KB revised the manuscript. All authors contributed to the article and approved the submitted version.

## Funding

This work was supported by Fonds voor Wetenschappelijk Onderzoek (FWO), Wetenschappelijk Fonds Willy Gepts (WFWG), and Strategic Research Programme (SRP48). KDV is a postdoctoral fellow of FWO (12I0921N).

## Conflict of interest

The authors declare that the research was conducted in the absence of any commercial or financial relationships that could be construed as a potential conflict of interest.

## Publisher’s note

All claims expressed in this article are solely those of the authors and do not necessarily represent those of their affiliated organizations, or those of the publisher, the editors and the reviewers. Any product that may be evaluated in this article, or claim that may be made by its manufacturer, is not guaranteed or endorsed by the publisher.
